# Burden of male hardcore smokers and its characteristics among those eligible for lung cancer screening

**DOI:** 10.1186/s12889-020-8266-z

**Published:** 2020-01-31

**Authors:** Dong Won Park, Ji-Yun Jang, Tai Sun Park, Hyun Lee, Ji-Yong Moon, Sang-Heon Kim, Tae-Hyung Kim, Ho Joo Yoon, Dae Ryong Kang, Jang Won Sohn

**Affiliations:** 1 Department of Internal Medicine, Hanyang University College of Medicine, 222-1 Wangsimni-ro, Seongdong-gu, Seoul, 04763 South Korea; 2 Department of Biostatistics, Yonsei University, Wonju College of Medicine, Wonju, Gangwon-do South Korea; 3 Center of Biomedical Data Science, Institute of Genomic Cohort, Yonsei University, Wonju College of Medicine, Wonju, Gangwon-do South Korea

**Keywords:** Lung cancer, Smokers, Smoking cessation, Cancer screening, Korea, Surveys

## Abstract

**Background:**

There are few data available about hardcore smokers and their behavioral characteristics among the lung cancer screening (LCS) population. The study investigated the burden of hardcore smokers within the LCS population, and determine the characteristics of hardcore smokers using nationally representative data in South Korea.

**Methods:**

We used data from 2007 to 2012 from the Korean National Health and Nutrition Examination Survey. This study enrolled current male smokers aged 55–74 years. Among them, subjects eligible for LCS were defined as these populations with smoking histories of at least 30 PY. Hardcore smoking was defined as smoking >15 cigarettes per day, with no plan to quit, and having made no attempt to quit. Multivariate logistic regression analyses were used to estimate associations between hardcore smokers and various sociodemographic and other variables.

**Results:**

The proportion of hardcore smokers among those who met LCS eligibility criteria decreased from 2007 to 2012 (from 39.07 to 29.47% of the population) but did not change significantly thereafter (*P* = 0.2770), and that proportion was consistently 10–15% higher than that of hardcore smokers among all male current smokers. The proportion without any plan to quit smoking decreased significantly from 54.35% in 2007 to 38.31% in 2012. However, the smokers who had made no intentional quit attempt in the prior year accounted for more than half of those eligible for LCS, and the proportion of such smokers did not change significantly during the study period (50.83% in 2007 and 51.03% in 2012). Multivariate logistic regression analyses showed that hardcore smokers were older (OR = 1.05, 95% confidence interval [CI] 1.01–1.09) than non-hardcore smokers. Hardcore smokers exhibited higher proportion of depression (OR = 6.55, 95% CI 1.75–24.61) and experienced extreme stress more frequently (OR = 1.93, 95% CI 1.13–3.29). Smokers who did not receive smoking cessation education within the past year were significantly more likely to be hardcore smokers (OR = 4.15, 95% CI 1.30–13.22).

**Conclusions:**

It is important to identify a subset of smokers unwilling or minimally motivated to quit within the context of lung cancer screening. Anti-smoking education should be enhanced to influence hardcore smokers’ behavior.

## Background

Lung cancer is the leading cause of cancer death in many countries, including Korea, representing a huge public health burden [1]. It is typically diagnosed at an advanced stage and long-term survival remains low [2]. Cigarette smoking is one of the most important predisposing factors [3]. Lung cancer screening (LCS) using low-dose computed tomography (LDCT) has been widely recommended to detect adult lung cancer at earlier treatable stages [4, 5]. Recently, the National Lung Screening Trial (NLST) showed that LDCT afforded a 20% reduction in the lung cancer mortality rate among high-risk adults aged 55–74 years with a smoking history of at least 30 pack-years (PY) and who were either current smokers or former smokers who had quit within the previous 15 years [6]. With these meaningful results of the NLST trial, the identification of well-defined populations at high risk of lung cancer makes the implementation of LCS a public health imperative. The combination of smoking abstinence and LCS also yielded the maximum reduction in mortality found in the NLST [7]. At the time of LCS, all current smokers should be advised to quit smoking, and former smokers should be advised to not resume [8].

Successful smoking cessation is essential within the context of LCS. As 48% of those who participated in the NLST were current smokers, they will constitute a large proportion of those undergoing LCS [6] Lung screening may constitute a teachable moment at which smoking cessation may be encouraged [9], but the evidence indicates that LCS via LDCT does not seem to increase smoking abstinence [10]. However, a major abnormality evident on LCS is significantly associated with higher rates of smoking cessation, and may be an important predictor of (less or no) subsequent smoking [11]. It is also of concern that a cancer-free LCS result may create false confidence among smokers, who then continue to smoke [12]. Previous population-based cancer screening programs have found that smoking and other unhealthy behaviors might be mutually reinforced after intervention at a teachable moment [13]. These findings encouraged us to investigate the smoking behaviors of LCS participants in an effort to attain successful smoking cessation.

Currently, tobacco control has reduced the prevalence of smoking in most countries. However, the rate of decline has slowed and certain subpopulations continue to smoke at disproportionately high rates [14]. In an effort to assist smokers resistant to tobacco controls, researchers have developed the concept of “hardcore smokers”; such smokers are less willing to quit, smoke heavily, and exhibit high-level nicotine dependence [15, 16]. Such characteristics predict future quitting attempts and smoking abstinence [17]. As a high-risk of lung cancer, the population undergoing LCS are older than the general population of smokers, are more likely to have medical comorbidities, and more likely to be heavy, long-standing smokers. Recent data show that current smokers eligible for LCS have varying levels of nicotine dependence, which predict both the quitting rate and clinical outcomes of lung cancer detection, and mortality [18]. Most studies of hardcore smoking have examined general populations [19, 20]. However, scarce researches have been conducted about the burden of hardcore smokers among the LCS population as a high risk group for nicotine dependence. Therefore, it is necessary to discuss the burden of hardcore smokers and their characteristics within the LCS population. This may allow LCS to be combined with smoking cessation interventions that improve LCS efficacy, which would improve the lung cancer mortality rate in public health.

This study investigated the burden of hardcore smokers within the Korean LCS population who met the LCS eligibility criteria by studying the proportion of hardcore smokers and its annual change, and identified the characteristics of hardcore smokers using nationally representative data.

## Methods

### Study population

We used data acquired by the Korean National Health and Nutrition Examination Survey IV–V (KHANES) from 2007 to 2012 to classify hardcore smokers. The Korean Ministry of Health and Welfare has performed the KNHANES since 1998. This is a cross-sectional nationally representative survey assessing the health and nutritional status of the non-institutionalized civilian population of South Korea [21]. Every year, the KNHANES extracts 23 households from each of 192 districts (via probabilistic sampling) and surveys about 10,000 household members aged 1 year or older. Sampling features two or three steps, and is stratified, clustered, and systematic based on sex, age, and geographic area as defined in household registries. The survey includes a health interview and health behavior, health examination, and nutrition surveys, which provide a variety of information on health status, health behavior, socioeconomic demographics; laboratory data are included.

Literature reported that LDCT for lung cancer has been reported to be effective screening method for lung cancer in high-risk populations, in large part due to the results of the NLST trial. The original eligibility criteria in the NLST trial included smokers aged 55–74 years with at least 30 PYs and current smoking status or having quit within the past 15 years. However, as the KNHANES survey items did not include the period of quitting smoking, the NLST eligibility criteria were not available for the former smokers. Thus, we enrolled only current smokers in the current study. Moreover, a previous study of a general Korean population found that males were fourfold more likely to be hardcore smokers than females [22]. Significant between-sex differences are evident in terms of workplace exposure, nicotine dependence, and access to healthcare [23]. In this regard, it was reasonable to focus on male hardcore smokers who met the NLST criteria for LCS. Therefore, in this study, we enrolled only current male smokers aged 55–74 years. Among them, subjects eligible for LCS were defined as these populations with smoking histories of at least 30 PY.

### Smoking status, definition of hardcore smoker, and features of hardcore smokers

Smokers were defined as respondents who had consumed ≥100 cigarettes over their lifetimes. Current smokers were those who smoked cigarettes “daily” or “sometimes,” and former smokers were those who did not smoke “now.” Never-smokers were those who had never consumed cigarettes. Previous researches has examined “hardcore” smokers, defined as smokers with low willingness to quit, heavy cigarette consumption, and high nicotine dependence [15]. Cigarettes per day is a measure of nicotine dependence used in the definition of hardcore smoking, and ranges from > 15 to > 25. Most studies agree that smokers can be classified as hardcore smokers if they smoke daily and smoke a minimum of 15 cigarettes per day [20, 22]. Similar to previous studies [16, 17, 24], we selected three characteristics of hardcore smokers related to continued smoking: (1) High daily cigarette consumption, defined as ≥15 cigarettes per day; (2) no intention of quitting, defined as never planning to quit in the past 6 months; and (3) having made no attempt to quit smoking that lasted for longer than 24 h in the past year. “Hardcore” smokers were defined as those who exhibited all three characteristics.

### Other variables

We recorded age, sex, body mass index, household income, educational attainment, occupation, marital status, and comorbidities. Age was classified into four groups: 55–59, 60–64, 65–69, and 70–74 years. Economic status was divided into the top and bottom two quartiles of household income. Educational attainment was divided into elementary school or lower, middle school, and high school or higher. Marital status was defined as married/cohabiting, single (divorced/widowed/separated), or never-married. We collected pulmonary function test (PFT) data, quality of life scores (obtained using the EuroQol five-dimension questionnaire [EQ-5D]), information on smoking-related factors (total amount of smoking, age at smoking initiation, exposure to secondhand smoke in the workplace/home, attendance at smoking cessation programs), alcohol use, physical activity, and psychological status. Age at smoking initiation was assessed using the information on smoking duration and age at the time of questionnaire completion. We divided age at smoking initiation into four groups (<16, 16–19, 20–25, and ≥ 26 years). We first considered grouping smokers by durations of 11–15, 26–30, and ≥ 31 years. However, because of a lack of sufficient numbers in these age groups, we collapsed these categories. Physical activity was defined as intense (jogging, mountain climbing, cycling, swimming rapidly, playing soccer or basketball, rope jumping, squash, or singles tennis for at least 20 min three times a week) or moderate (including swimming slowly; and playing doubles tennis, volleyball, badminton, or table tennis). Muscle-strengthening exercises included push-ups, sit-ups, dumbbell or barbell exercises, or work on a horizontal bar. The variables used to assess psychological status explored stress as follows: “How stressed do you feel during your normal days?” One of the following responses to this question (“extremely”, “sufficiently”, “hardly” or “a little”) was used.

### Statistical analyses

We analyzed the data using a complex sample design that considers the data characteristics. Weighted values used in the surveys of the 6-year period were combined into single values and a plan file created. We analyzed the data in two ways. First, we used linear-by-linear association to examine the 6-year trends in the age-adjusted proportion of hardcore smokers and measures taken to cease hardcore smoking. Second, hardcore smokers and others were compared in terms of age, sex, body mass index, sociodemographic factors, underlying disease, pulmonary function test (PFT) results, stress level, smoking-related factors, alcohol use, EQ-5D scores, and physical activity. Variables with *P*-value ≤0.20 in univariate analyses were entered into a multivariate logistic regression analysis. We evaluated the variance inflation factor and found no multicollinearity among the independent variables. The results are described as odds ratios (ORs) with 95% confidence intervals. Statistical analyses were performed using SAS Enterprise Guide ver. 9.4 software (SAS Institute, Cary, NC, USA). A *P*-value <0.05 was deemed to be statistically significant.

### Ethics statement

The Korea Centers for Disease Control and Prevention obtained written informed consent from all KNHANES participants. After registering personal information and signing a pledge of confidentiality, anyone can download raw data from the KNHANES website. As the datasets are publicly available, ethics approval was not required. The protocol was approved by the institutional review board (IRB) of Hanyang University Hospital, Seoul, South Korea (IRB No. 2018–12–014).

## Results

Of those who were surveyed from 2007 to 2012, we identified 1551 current male smokers aged 55–74 years of whom 891 had histories of more than 30 PY of heavy smoking and met the Korean, male, LCS criteria. As the weighted value used in this study, a total of 4,763,098 males were obtained during the 6-year period.

### Annual changes in hardcore smoker proportions and characteristics

**Fig. 1:**
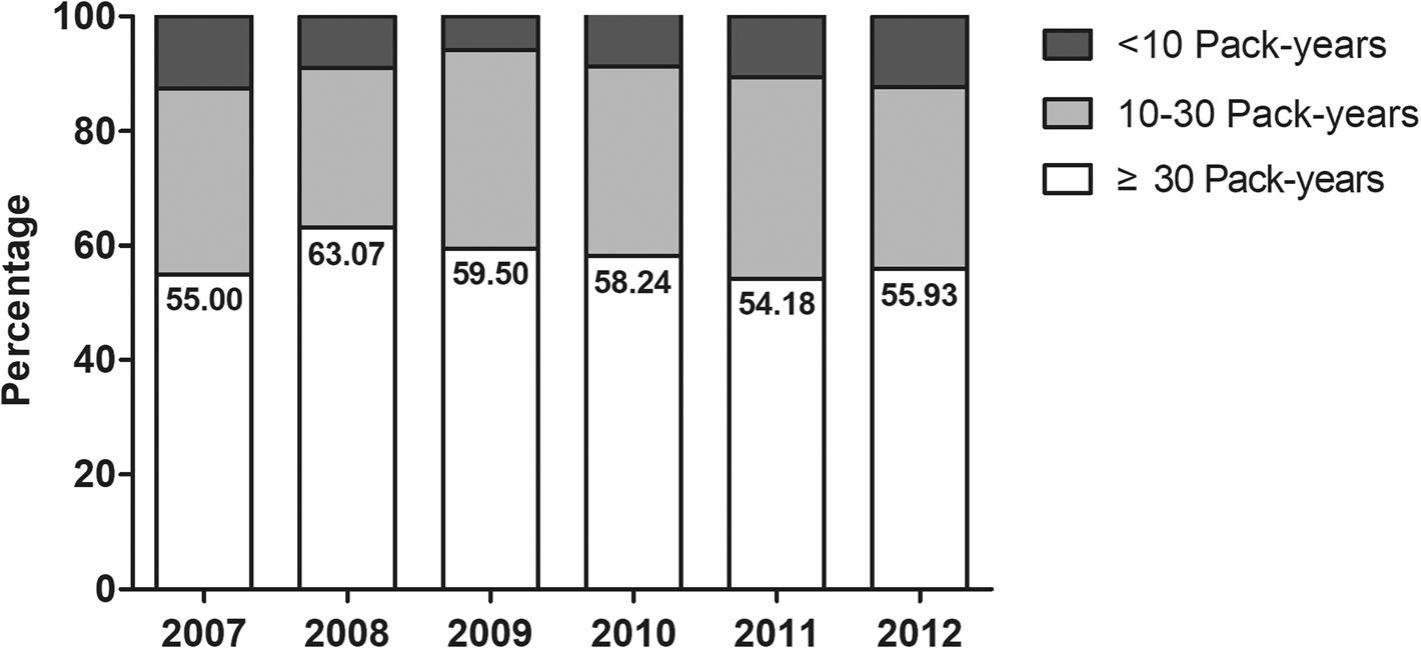
The annual percentages of male current smokers aged 55–74 years by the extent of smoking

Table 1 shows the weighted proportion of hardcore smokers and their characteristics among current male smokers aged 55–74 years. The proportion of male current smokers with histories of more than 30 PY was 55–60% among all male current smokers (being 55.0% in 2007 and 55.93% in 2013, Fig. 1), and the weighted proportion of such males did not decrease from 2007 to 2012 (Fig. 1), being maintained in all male current and former smokers in this age group (Additional file 1: Figure S1). Among all current smokers, about 60% of all current smokers aged 55–74 years smoked more than 15 cigarettes per day, and hardcore smokers accounted for about 17–22% of these. However, almost all smokers with histories of more than 30 PY smoked more than 15 cigarettes per day during the study period (being 99.14% in 2007 and 97.41% in 2012). Although the proportion stating “no plan to quit” decreased from 2007 to 2012 (from 54.35 to 38.31%, P_trend_ = 0.0382), the proportion reporting “no quit attempt made in the past 12 months” did not change significantly (being 50.83% in 2007 and 51.03% in 2012); the trend was similar for all current smokers. The proportion of hardcore smokers among current smokers with more than 30 PYs decreased from 2007 to 2012 (from 39.07 to 29.47%) but the trend did not change significantly (P_trend_ = 0.2770). That proportion was consistently 10–15% higher than that of hardcore smokers among all male current smokers.

**Table 1:** Weighted proportions of hardcore smokers, and their characteristics, among current male smokers aged 55–74 years old

Hardcore smoking measures	2007	2008	2009	2010	2011	2012
All current smokers						
High daily cigarette consumption	56.93	66.29	64.70	65.18	63.71	58.14
No plan to quit*	50.00	46.98	39.22	33.06	29.64	34.57
No quit attempt in the past 12 months	46.16	50.58	46.39	48.31	45.20	43.81
Hardcore smoker **	22.61	26.22	18.50	19.19	18.87	17.01
Current smoker ≥30 Pack-years						
High daily cigarette consumption	99.14	99.62	99.70	96.55	99.30	97.41
No plan to quit†	54.35	52.09	42.74	39.06	37.94	38.31
No quit attempt in the past 12 months	50.83	59.57	54.52	58.22	55.61	51.03
Hardcore smoker††	39.07	40.95	30.32	30.23	31.55	29.47

*P <  0.0001 for trend for smokers without plan to quit among all male current smoker; **P = 0.2966 for trend for hardcore smokers among all male current smoker; †*P* = 0.0382 for trend for smokers without plan to quit among male current smoker (≥ 30 Pack-years); ††*P* = 0.2770 for trend for hardcore smokers among male current smoker (≥ 30 Pack-years)

### Baseline characteristics of the study subjects

The demographic and clinical characteristics of the study population are shown in Table 2. Compared to non-hardcore smokers, hardcore smokers were older, had a lower BMI, were less educated, and had a higher proportion of depression. However, the two groups did not differ significantly in terms of occupation, marital status or the proportion of obstructive PFT patterns. As shown in Table 3, an early age at smoking commencement, a high level of smoking, little education on smoking cessation, and high alcohol consumption were significantly associated with hardcore smoker status. A large proportion of such smokers did not engage in intense or moderate physical activity or muscle-strengthening exercises, compared to non-hardcore smokers. Although the EQ-5D revealed no significant difference in the quality of life, the self-perceived stress level was higher in hardcore than non-hardcore smokers.

**Table 2:** Baseline characteristics of hardcore smokers among current male smokers aged 55–74 years old with histories of more than 30 pack-years of smoking

Variable		All (*N* = 4,763,098)	Hardcore smoker (*N* = 1,576,339)	Non-hardcore smoker (*N* = 3,186,759)	*P*-value
Age	mean ± SD	62.88 ± 0.20	63.91 ± 0.35	62.37 ± 0.25	0.0006
	55 ≤ age < 60	1,487,173 (31.23)	375,438 (23.82)	1,111,735 (34.89)	0.0052
	60 ≤ age < 65	1,557,224 (32.69)	507,820 (32.22)	1,049,403 (32.93)	
	65 ≤ age < 70	1,016,798 (21.35)	377,478 (23.95)	639,320 (20.06)	
	70 ≤ age < 75	701,902 (14.74)	315,602 (20.02)	386,300 (12.12)	
BMI, kg/m2	mean ± SD	23.14 ± 0.11	22.82 ± 0.21	23.30 ± 0.13	0.0487
Household income	1st quartile	1,548,917 (33.24)	600,812 (39.40)	948,105 (30.25)	0.1380
	2nd quartile	1,287,828 (27.64)	391,740 (25.69)	896,088 (28.59)	
	3rd quartile	1,034,921 (22.21)	315,490 (20.69)	719,431 (22.95)	
	4th quartile	787,801 (16.91)	216,823 (14.22)	570,978 (18.22)	
Education	Elementary school or lower	1,984,579 (41.79)	780,634 (49.61)	1,203,944 (37.91)	0.0149
	Middle school	1,083,487 (22.81)	328,611 (20.88)	754,877 (23.77)	
	High school or higher	1,681,244 (35.40)	464,265 (29.51)	1,216,979 (38.32)	
Occupation	White collar job	238,122 (5.03)	55,322 (3.55)	182,799 (5.76)	0.3717
	Blue collar job	2,972,211 (62.84)	973,748 (62.53)	1,998,463 (62.99)	
	Unemployed	1,519,481 (32.13)	528,295 (33.92)	991,186 (31.24)	
Marriage	Married/cohabiting	4,253,914 (90.30)	1,377,576 (88.43)	2,876,338 (91.23)	0.2296
	Single, never-married	456,756 (9.70)	180,293 (11.57)	276,463 (8.77)	
Underlying disease	Hypertension	1,482,919 (31.13)	534,463 (33.91)	948,456 (29.76)	0.2519
	Diabetes mellitus	929,758 (19.52)	297,874 (18.90)	631,884 (19.83)	0.7799
	Cardiovascular disease	296,749 (6.23)	94,423 (5.99)	202,326 (6.35)	0.8715
	Dyslipidemia	556,939 (11.69)	165,662 (10.51)	391,277 (12.28)	0.5366
	Cerebral vascular accident	172,143 (3.61)	69,577 (4.41)	102,566 (3.22)	0.4451
	Depression	64,651 (1.36)	41,320 (2.62)	23,332 (0.73)	0.0240
FEV1/FVC	< 0.7	1,572,220 (43.43)	535,954 (46.77)	1,036,267 (41.88)	0.3029
	≥ 0.7	2,048,217 (56.57)	610,074 (53.23)	1,438,143 (58.12)	

Data are presented as either means ± SD or as numbers and percentages, as appropriate. *SD* Standard deviation, *PY* Pack-year; Single includes divorced, bereaved, separated; Chronic disease includes hypertension, cardiovascular disease, diabetes mellitus; *FEV1* Forced expiratory volume in 1 sec, *FVC* Forced vital capacity

**Table 3:** Smoking, alcohol use, physical activity level, and the quality of life of hardcore smokers among current male smokers aged 55–74 years old with histories of more than 30 pack-years of smoking

Variable	All (*N* = 4,763,098)	Hardcore smoker (*N* = 1,576,339)	Non-hardcore smoker (*N* = 3,186,759)	*P*-value
Smoking history				
Age at smoking initiation				
mean ± SD, years	20.14 ± 0.15	19.91 ± 0.29	20.26 ± 0.18	0.3181
age < 16	448,900 (9.42)	96,125 (12.44)	252,775 (7.93)	0.1983
16–20	1,558,601 (32.72)	473,733 (30.05)	1,084,869 (34.04)	
20–26	2,354,200 (49.43)	793,997 (50.37)	1,560,203 (48.96)	
≥ 26	401,396 (8.43)	112,484 (7.14)	288,912 (9.07)	
Total amount of smoking (mean ± SD, PYs)	45.94 ± 0.59	48.71 ± 1.06	44.57 ± 0.65	0.0008
Second-hand smoke at workplace	2,215,002 (59.65)	783,208 (62.85)	1,431,794 (58.03)	0.2882
Second-hand smoke at home	534,864 (60.82)	205,697 (57.65)	329,167 (62.99)	0.5190
Education on smoking cessation	295,023 (6.19)	31,159 (1.98)	263,864 (8.28)	0.0012
Alcohol use				0.0250
Never drinker	603,562 (13.38)	207,816 (13.94)	395,745 (13.10)	
0–1 time per week	1,402,721 (31.09)	383,625 (25.73)	1,019,097 (33.74)	
2–3 times per week	1,154,395 (25.58)	337,568 (22.64)	816,827 (27.04)	
≥ 4 times per week	1,351,464 (29.95)	562,243 (37.70)	789,221 (26.13)	
Physical activity (days per week)				
Intense physical activity				<0.0001
None	3,455,345 (72.62)	1,196,667 (76.15)	2,258,677 (70.88)	
1 day or more	1,302,777 (27.38)	374,696 (23.85)	928,081 (29.12)	
Moderate physical activity				0.0922
None	3,207,816 (67.46)	1,124,207 (71.54)	2,083,608 (65.44)	
1 day or more	1,547,407 (32.54)	447,156 (28.46)	1,100,251 (34.56)	
Walking				0.4154
None	982,587 (20.63)	354,400 (22.48)	628,187 (19.71)	
1 day or more	3,780,510 (79.37)	1,221,939 (77.52)	2,558,571 (80.29)	
Muscle strengthening exercises				0.0062
None	3,576,412 (75.13)	1,288,433 (81.74)	2,287,979 (71.86)	
1 day or more	1,183,787 (24.87)	287,906 (18.26)	895,881 (28.14)	
EQ-5D				
Mobility				0.1598
No problem	3,593,648 (75.67)	1,142,042 (72.58)	2,451,606 (77.20)	
Problem	1,155,661 (24.33)	431,468 (27.42)	724,193 (22.80)	
Self-care				0.7847
No problem	4,394,378 (92.52)	1,462,178 (92.92)	2,932,200 (92.33)	
Problem	354,932 (7.47)	111,332 (7.08)	243,600 (7.67)	
Usual activities				0.1462
No problem	4,074,653 (85.79)	1,309,170 (83.20)	2,765,483 (87.08)	
Problem	674,656 (14.21)	264,340 (16.80)	410,316 (12.92)	
Pain/discomfort				0.7049
No problem	3,526,198 (74.25)	1,153,523 (73.31)	2,372,675 (74.71)	
Problem	1,223,111 (25.75)	419,987 (26.69)	803,125 (25.29)	
Anxiety/depression				0.4414
No problem	4,093,048 (86.18)	1,332,971 (84.71)	276,007 (86.91)	
Problem	656,262 (13.82)	240,539 (15.29)	415,723 (13.09)	
Stress recognition				0.0494
Hardly or a little	1,047,737 (22.00)	303,937 (19.28)	743,800 (23.34)	
Sufficiently	2,563,226 (53.81)	802,693 (50.92)	1,760,533 (55.25)	
Extremely	1,152,135 (24.19)	469,710 (29.80)	682,425 (21.41)	

Data are presented as either means ± SD or as numbers and percentages, as appropriate. *SD* standard deviation; *PY* Pack-year; *EQ-5D* EuroQol five-dimension questionnaire

### Characteristics of hardcore smokers

Table 4 shows the results of multivariate logistic regression analyses revealing the parameters contributing to the likelihood of hardcore smoking. Hardcore smokers were older (OR = 1.05, 95% CI 1.01–1.09), and exhibited a higher proportion of depression (OR = 6.55, 95% CI 1.75–24.61) compared with non-hardcore smokers. Notably, compared to smokers who had been educated on smoking cessation, those lacking such education within the past year were significantly more likely to be hardcore smokers (OR = 4.15, 95% CI 1.30–13.22). Hardcore smokers were nearly two times more frequent among smokers under extreme stress than among those under little stress (OR = 1.93, 95% CI 1.13–3.29).

**Table 4:** Multivariate logistic regression analyses of data on hardcore smokers among male current smokers aged 55–74 years old with histories of more than 30 pack-years of smoking, and their characteristics

Variable	Hardcore smoker OR (Univariate)	Hardcore smoker OR (Multivariate)
Baseline characteristics		
Age, years	1.06 (1.02–1.09)	1.05 (1.01–1.09)
BMI, kg/m^2^	0.95 (0.90–1.00)	0.99 (0.93–1.05)
Underlying disease		
Depression (vs. none)	3.65 (1.09–12.23)	6.55 (1.75–24.61)
Chronic diseases (vs. none)	1.03 (0.71–1.34)	
Household income (vs. 4th quartile)		
1st quartile	1.67 (0.99–2.82)	1.03 (0.58–1.84)
2nd quartile	1.15 (0.65–2.03)	0.78 (0.41–1.46)
3rd quartile	1.16 (0.66–2.03)	0.98 (0.54–1.80)
Education (vs. high school or higher)		
Elementary school or lower	1.70 (1.15–2.51)	1.50 (0.98–2.30)
Middle school	1.14 (0.74–1.77)	0.96 (0.59–1.56)
Occupation (vs. white collar job)		
Blue collar job	1.61 (0.79–3.29)	
Unemployed	1.76 (0.83–3.74)	
Marital status		
Single (vs. married/ cohabiting)	1.36 (0.82–2.26)	
FEV1/FVC (vs. ≥ 0.7)		
FEV1/FVC < 0.7	1.22 (0.84–1.78)	
Smoking and alcohol related factors		
Age at smoking initiation (vs. ≥26 years)		
age < 16 years	1.99 (0.95–4.19)	2.08 (0.89–4.85)
16–20 years	1.12 (0.58–2.18)	1.13 (0.52–2.46)
20–26 years	1.31 (0.70–2.43)	1.51 (0.71–3.17)
Second-hand smoke at workplace (vs. none)	1.22 (0.84–1.78)	
Second-hand smoke at home (vs. none)	0.80 (0.39–1.66)	
No education on smoking cessation (vs. yes)	4.78 (1.69–11.87)	4.15 (1.30–13.22)
Alcohol use (vs.never-drinker)		
0–1 time per week	0.72 (0.39–1.33)	0.96 (0.49–1.90)
2–3 times per week	0.79 (0.42–1.48)	1.04 (0.49–2.19)
≥ 4 times per week	1.36 (0.77–2.38)	1.73 (0.91–3.27)
Life style factors		
Stress recognition (vs. hardly or a little)		
Sufficiently	1.12 (0.73–1.72)	1.37 (0.85–2.22)
Extremely	1.68 (1.06–2.69)	1.93 (1.13–3.29)
Number of days of intense physical activity		
None (vs. ≥ 1 day, per week)	1.31 (0.90–1.92)	0.94 (0.60–1.48)
Moderate physical activity		
None (vs. ≥ 1 day, per week)	1.33 (0.95–1.85)	1.16 (0.78–1.71)
Walking		
None (vs. ≥ 1 day, per week)	1.18 (0.79–1.77)	
Muscle strengthening exercises		
None (vs. ≥ 1 day, per week)	1.75 (1.17–2.63)	1.32 (0.82–2.12)
EQ-5D		
Mobility problems (vs. none)	1.28 (0.91–1.80)	1.05 (0.63–1.75)
Self-care problems (vs. none)	0.92 (0.49–1.72)	
Usual activity problems (vs. none)	1.36 (0.90–2.07)	1.06 (0.56–2.00)
Pain/discomfort problems (vs. none)	1.08 (0.74–1.57)	
Anxiety/depression problems (vs. none)	1.20 (0.76–1.90)	

Single includes divorced, bereaved, separated, and never-married; chronic disease includes hypertension, cardiovascular disease, diabetes mellitus. *EQ-5D* EuroQol five-dimension questionnaire, *FEV1* Forced expiratory volume in 1 sec, *FVC* Forced vital capacity

## Discussion

Our study found that the proportion of smokers with histories of more than 30 PY was 55–60% among all male current smokers aged 55–74 years. Almost all smokers with histories of more than 30 PY smoked more than 15 cigarettes per day during the study period. More than half of such smokers responded “no quit attempt made in the past 12 months”. Moreover, more than one third of male current smokers with more than 30 PY were hardcore smokers, and that proportion was consistently 10–15% higher than that of hardcore smokers among all male current smokers. This study also demonstrated significant associations between male Korean hardcore smokers eligible for LCS and older age, higher prevalence of depression, higher level of stress, and lack of education regarding smoking cessation.

Most studies of hardcore smoking have examined general populations. Although the prevalence has differed with the definition used and the state of the tobacco epidemic, the literature on general populations implies that hardcore smokers comprise a minority of smokers [25]. Studies with similar definitions of hardcore smokers have found that they constitute 5.2% of Californian [26] and 9.7% of Italian [27] smokers. A recent Korean study of smokers aged 19 or older found that about 20% of smokers are hardcore [22]. However, these findings might be influenced by the proportion of middle-aged individuals (30–59 years) with high prevalence rates. Considering these reports, we found a consistently higher proportion of hardcore smokers among LCS participants, implying a significant impact of hardcore smokers among subjects eligible for LCS. To our knowledge, the prevalence of hardcore smokers in the LCS population has not been studied, although their behavioral characteristics have been described. Of the previous report on NLST population, 55% were current smokers, and 57.4% of these had made no intentional quit attempt in the past year [28]. In Dutch–Belgian, randomized, controlled, lung cancer screening trial, lung cancer screening trial, 40% of the respondents had no intention of quitting smoking in the near future (within about 1 year) [29]. These proportions of smokers unwilling or unmotivated to quit smoking among LCS-eligible subjects are consistent with our results.

Notably, the annual proportion of hardcore smokers among LCS participants did not decrease over the study period. Most recent evidence does not support the hardening hypothesis in general populations, consistently showing either no change in hardcore smoking measures or even a decrease in hardcore smokers [30, 31]. Moreover, specifically in Korea, nationwide smoking cessation programs (including specific clinics) were introduced by the Korean government in 2005 and a toll-free telephone “quit-line” in 2006, associated with 253 healthcare clinics that provide free nicotine replacement therapy and individual counseling before the study period. The price of cigarettes increased almost twofold in 2015 during the study period. In a previous study on hardcore smokers in the general, Korean adult population, the prevalence of current smokers decreased from 2001 (25.0%) to 2012 (13.0%) [22]. These results might be related to the efforts increasing quitting rate among smokers in public. However, even the effort to increase the quitting rate among smokers in Korea, has not eased the burden that hardcore smokers place on the LCS population. High-level nicotine dependence has frequently been cited as a characteristic of hardcore smokers [15]. Rojewski et al. [18] showed that high nicotine dependence was significantly related to the lung cancer risk and lung cancer-specific mortality in the LCS population. This suggests that prompt detection of such hardcore smokers allow to increase the benefit of the LCS program. Our findings contribute to the literature, showing that hardcore smokers are widespread and continue to remain among LCS participants. These are the main features of this study that deserve to be highlighted.

This present study also showed that the proportion without a plan to quit decreased significantly from 54.35% in 2007 to 38.31% in 2012, however the proportion who did not attempt to quit in the past year accounted for more than half and did not change significantly over the study period. Increasing numbers of smokers who meet the LCS criteria plan to stop smoking. Nevertheless, more than half in our study did not attempt to quit, emphasizing the difference between planning and action. Instituting smoking cessation requires changes in knowledge, attitudes, and beliefs about smoking [32]. LCS creates an additional interaction between smokers and healthcare providers, affording a teachable moment that can be used to promote the desire to change [10]. A recent study on a subset of NLST participants described the association between the clinician-delivered “5As” (Ask, Advise, Assess, Assist, and Arrange) and quitting behavior [33]. Of the 5As, assisting and arranging (in terms of NLST follow-up) were associated with increased quitting rates (OR 1.40 and OR 1.26, respectively), however, these rates remained relatively low. Our study showed that smokers who did not receive education on smoking cessation within the past year were significantly more likely to be less willing to quit smoking. Firm evidence exists that smoking cessation services should be combined with LCS [34–36]. Effective smoking education and interventions enhance the benefits afforded by LCS, reducing mortality and morbidity. Recent collaborative trials have considered these issues and will develop better smoking cessation strategies [35, 37]. Our results underline the importance of enhancing anti-smoking education to influence the behaviors of hardcore smokers among LCS participants.

Identification of characteristics associated with failure to abstain and successful quitting may facilitate the development of more effective smoking cessation interventions for LCS participants. Here, we evaluated the characteristics of hardcore smokers eligible for LCS. The literature on the association between age and hardcore smoking in the general population is mixed. Some reports have found a high proportion of persistent smokers and low rates of decline in smoking prevalence in persons over 65 years old [31], and that older age groups had the lowest intention to quit [38]. However, younger men are more likely to be long-term smokers and less likely to quit than their older counterparts [39]. We found that hardcore smoking was more prevalent among older age group in smokers aged 55–74 years eligible for LCS. In addition, the age at smoking initiation is negatively correlated with persistent smoking [26]. In this study, onset of smoking at a younger age (< 16 years old) was positively associated with the likelihood of hardcore smoking, but no statistical significance was noted (OR = 2.08, CI 0.89–4.85).

We also found that hardcore smokers had a higher proportion of depression. Mental illness, such as major depression and anxiety disorders, increases smoking rates, and smokers with mental illness have more difficulty quitting than do those without mental illness [40, 41]. The readiness to quit smoking among smokers with mental illness appears to be related to nicotine dependence [42, 43], which is consistent with the finding that nicotine dependence mediates the associations among anxiety, depression, and smoking maintenance [44] (Morrell, Holly E. R. 2006). Cinciripini et al. [45] suggested that a depressed mood after an attempt to quit smoking relates to continued smoking and poor quitting self-efficacy. Significant association has been observed between depressive disorders and hardcore smoking, in line with our results. In addition, we showed that a high level of stress was prevalent among hardcore smokers. In line with our findings, Daoud et al. [46] reported that psychological factors including anxiety and stressful life events are correlated with quitting behaviors. A high level of perceived stress is associated with hardcore smoking [27] and low success with cessation [47]. However, the questionnaire used in this study might have been too simplistic to accurately evaluate the severity of depression or stress. Therefore, further research is needed to address the impact of psychological factors on hardcore smoking and quitting behaviors.

Our study results showed no significant differences in the frequency of spirometric airflow obstruction between hardcore and non-hardcore smokers. Chronic obstructive pulmonary disease (COPD) and lung cancer often coexist, and they share the risk factor of cigarette smoking. COPD is also associated with an increased risk of lung cancer in the setting of LCS [48]. One study on smokers with COPD reported that nearly half were not planning to quit [49], and a significant association was noted between COPD severity and quitting success [50]. The absence of disparities in COPD frequency among hardcore smokers suggests that the population eligible for LCS might smoke a considerable amount regardless of hardcore smoking. Moreover, we postulate that the severity of COPD was not reflected in this study and the participating groups included patients with relatively mild COPD due to the nature of the KNHANES investigation on adult health and nutritional conditions.

Our study had certain limitations. First, this study did not cover the actual population of those undergoing LCS, although all members of our population were LCS candidates. If LDCT screening yields abnormal results, this may create a teachable moment for LCS participants who are considering to quit smoking. A concern of poor lung health may motivate smokers to quit, which would affect the proportion of hardcore smokers. Second, no standard definition of hardcore smoking is available; the conceptual definitions have included nicotine-dependence, no intention to quit, poor self-efficacy, and lack of motivation [15]. We used only three characteristics to define hardcore smoking because we lacked data on other variables such as the extent of nicotine dependence. Third, difficulty confirming the causal relationship between hardcore smokers and various characteristics in LCS screening population because this was an observational cross-sectional study. Fourth, we focused on current male smokers; thus, our results may not be generalizable to former male smokers or female smokers.

## Conclusions

We found that more than one third of male current smokers eligible for LDCT cancer screening were hardcore smokers, and that proportion does not show a downward annual trend. Compared to non-hardcore smokers, hardcore smokers eligible for LCS are older, exhibit a higher proportion of depression, and experience extreme stress more frequently. Smokers who have not received smoking cessation education within the past year were significantly more likely to be hardcore smokers. Although intensive smoking cessation policies cover the entire LCS population, it is important to identify the specific groups with higher prevalence of hardcore smokers in the setting of LCS and to strengthen the comprehensive smoking cessation strategies by targeting these vulnerable populations. Future stop-smoking interventions should consider these characteristics of the population.

## Supplementary information

**Additional file 1: Figure S1.** The annual percentages of all male current and former smokers aged 55–74 years by the extent of smoking.

## Data Availability

The dataset we used for the research, detailed information on the survey design and characteristics are provided on the KNHANES homepage (https://knhanes.cdc.go.kr/knhanes/eng/index.do).

## Abbreviations

COPD: Chronic obstructive pulmonary disease
EQ-5D: EuroQol five-dimension questionnaire
IRB: Institutional review board
KHANES: Korean National Health and Nutrition Examination Survey
LCS: Lung cancer screening
LDCT: Low-dose computed tomography
NLST: National Lung Screening Trial
OR: Odds ratios
PFT: Pulmonary function test
PY: Pack-years
