# Supplementary figures and images for: Burden of male hardcore smokers and its characteristics among those eligible for lung cancer screening
Source: BMC Public Health. 2020 Jan 31;20:151. doi: 10.1186/s12889-020-8266-z (PMC6995174; doi:10.1186/s12889-020-8266-z)

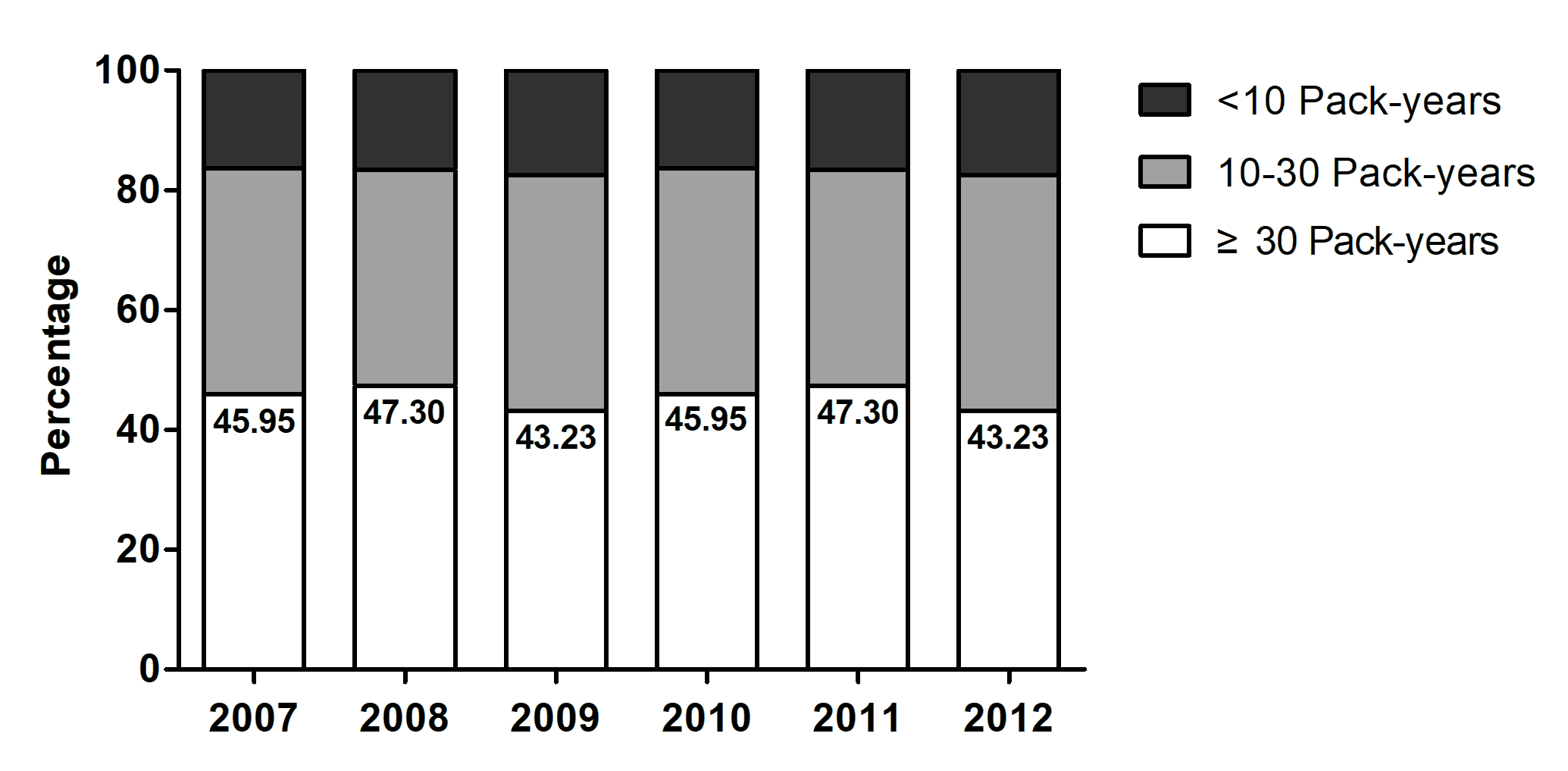

Supplement: Supplementary file 1 — Additional file 1: Figure S1. The annual percentages of all male current and former smokers aged 55–74 years by the extent of smoking. [file 12889_2020_8266_MOESM1_ESM.tif]
